# Concurrent reactive arthritis, Graves’ disease, and warm autoimmune hemolytic anemia: a case report

**DOI:** 10.4076/1757-1626-2-6988

**Published:** 2009-08-13

**Authors:** Elizabeth Chiang, Clifford D Packer

**Affiliations:** 1 Department of Neuroscience, Case Western Reserve University School of Medicine10900 Euclid Avenue, Cleveland, OH 44106USA; 2 Department of Internal Medicine, Louis Stokes Cleveland Veterans Affairs Medical Center10701 East Boulevard, Cleveland, OH 44106USA

## Abstract

Warm antibody autoimmune hemolytic anemia is due to the presence of warm agglutinins that react with protein antigens on the surface of red blood cells causing premature destruction of circulating red blood cells. We report the first case of concurrent reactive arthritis, Graves’ disease, and autoimmune hemolytic anemia. A 40-year-old man with reactive arthritis, Graves’ disease, type 2 diabetes mellitus, mitral valve prolapse, and Gilbert’s disease presented with a one month history of jaundice, fatigue, and black stools. After diagnosis of warm autoimmune hemolytic anemia, the patient was started on prednisone 1 mg/kg with rapid improvement in his anemia and jaundice. Our subject’s mother and possibly his maternal grandmother also had autoimmune hemolytic anemia, which raises the possibility of hereditary autoimmune hemolytic anemia, a rarely reported condition.

## Introduction

Autoimmune hemolytic anemia is occasionally reported in patients with other autoimmune illnesses, most commonly systemic lupus erythematosis, rheumatoid arthritis, scleroderma, and ulcerative colitis [1]. There are at least 10 case reports of autoimmune hemolytic anemia associated with hyperthyroidism [2-11], and 1 report of autoimmune hemolytic anemia associated with reactive arthritis [12]. However, a Medline search reveals no previous reports of concurrent reactive arthritis, hyperthyroidism, and autoimmune hemolytic anemia. We report the case of a 40-year-old man who developed severe warm autoimmune hemolytic anemia while under treatment for both Graves’ disease and reactive arthritis. Our subject’s mother and possibly his maternal grandmother also had autoimmune hemolytic anemia, which raises the possibility of hereditary autoimmune hemolytic anemia, a rarely reported condition [13-16].

## Case presentation

A 40-year-old Caucasian American man with reactive arthritis, Graves’ disease, type 2 diabetes mellitus, mitral valve prolapse, and Gilbert’s disease was admitted with one month of progressive jaundice, fatigue, lightheadedness, and exertional dyspnea. He also described dark urine (“the color of raspberry iced tea”) and dark brown to black stools. He denied fevers or chills, but gave a long history of night sweats and occasional diarrhea, which he ascribed to his medications. He had no history of blood transfusions, prior hepatitis, alcohol use, intravenous drug use, or tattoos, and he did not use herbal medications or supplements. He had recently come to Cleveland to help care for his father, who had stage IV colon cancer. His mother had been diagnosed with autoimmune hemolytic anemia at the age of 40; she was treated with corticosteroids and eventually required splenectomy. His maternal grandmother also had anemia and jaundice, although the patient was not aware of the cause. His medications were etanercept, metformin, pioglitazone, methimazole, niacin, and aspirin. He had stopped the pioglitazone and metformin more than one month prior to admission (before the onset of jaundice) on the advice of an endocrinologist. He had been diagnosed with reactive arthritis about 10 years before admission, and had been treated with etanercept for the previous 8 years. His Graves’ disease had been diagnosed 18 months before admission, and treated over that time with methimazole.

Physical examination revealed a calm, well-nourished man with scleral icterus and generalized jaundice. Blood pressure was 130/76, heart rate 102/min., respiratory rate 16/min, temperature 97.7F. There was no cervical, supraclavicular, epitrochlear, axillary, or inguinal lymphadenopathy. The thyroid gland was not enlarged or tender, and there was no proptosis, lid-lag, or tremor. The lungs were clear and the heart rhythm was regular without murmur, click, or gallop. The abdomen was soft and non-tender, with the liver edge palpable 2 cm below the right costal margin; the spleen was not palpable. A vesiculobullous rash was seen on the plantar aspect of the right foot.

Laboratory tests were significant for hemoglobin 5.8 g/dL, hematocrit 18.7%, MCV 107.5, platelet count 231,000, WBC count 9,800, reticulocyte count 23.4%, Bilirubin 13.6 (direct 0.6), LDH 369, and haptoglobin <6. Hepatitis A, B, and C serologies, antinuclear antibody, antimicrosomal antibody, D-dimer, cold agglutinins, cryoglobulins, and HIV test were negative. The fibrinogen was normal at 342. The INR was 0.8 and the partial thromboplastin time was 26.4. The peripheral smear showed spherocytes and bite cells, with no schistocytes, helmet cells, spur cells, sickle cells, or teardrop cells. The differential was 54.1% neutrophils, 35.6% lymphocytes, 8.3% monocytes, 1.7% eosinophils, and 0.3% basophils. Direct antiglobulin test (DAT) was + for anti IgG and negative for anti-C3. The indirect antiglobulin test was negative. The G6PD level was normal. TSH was 1.067.

Lumbar spine X-rays showed ankylosis of the sacroiliac joints. Computed tomographic scan of the chest, abdomen and pelvis showed probable enlarged thymus in the anterior mediastinum, a mildly enlarged spleen, and tiny bilateral renal and hepatic hypodensities too small to be characterized.

The patient was started on prednisone 1 mg/kg with rapid improvement in his anemia and jaundice. 11 days after admission his hemoglobin has improved to 10.0 g/dL. 29 days after admission, the hemoglobin was 14.7 and the bilirubin had decreased to 3.6 mg/dL. The prednisone was tapered off over 3 months with continued stable hemoglobin levels and no evidence of recurrent hemolysis.

## Discussion

Hemolytic anemia is caused by premature destruction of circulating red blood cells. It can be intravascular, as in microangiopathy, infections, transfusion reactions, and paroxysmal cold hemoglobinuria, or extravascular, as in enzyme deficiencies, membranopathies, hemoglobinopathies, drug reactions, and most autoimmune hemolytic anemias.

In this case, the elevated LDH and indirect bilirubin, low haptoglobin, and high reticulocyte count all were consistent with hemolysis. There was no fever, leukocytosis, or pertinent travel history to suggest malaria or babesiosis, and there was no evidence of spider or insect bites. The patient did not have a history of chronic anemia, which was against the diagnosis of hereditary spherocytosis. The normal fibrinogen, negative D-dimer, and absence of schistocytes on peripheral smear ruled out a microangiopathic process. A normal G6PD level, obtained after the acute phase of the illness, eliminated G6PD deficiency as a possibility. The DAT revealed an anti-IgG antibody on the RBC’s, consistent with either a warm autoimmune hemolytic anemia (WAIHA) or a drug-induced hemolytic anemia (DIHA). There are 4 case reports of metformin-induced hemolytic anemia [17], but our patient had stopped metformin at least a month before his symptoms developed, so this was very unlikely. There is one reported case of a patient with rheumatoid arthritis who developed cold agglutinin disease during etanercept treatment [18], but cold agglutinin disease is associated with IgM antibodies and typically causes only mild hemolysis. Our patient’s cold agglutinin screen was negative. None of his other medications have been reported to cause hemolytic anemia. The leading diagnosis was therefore WAIHA. Most cases of WAIHA are idiopathic, but secondary causes can include viral infections, autoimmune disorders (particularly SLE, RA, UC, and scleroderma), immune system malignancies (most commonly CLL), AIDS and other immunodeficiency disorders, and various non-lymphoid neoplasms. The work-up for secondary causes was negative with the exception of the CT scan findings of thymus enlargement, mild splenomegaly, and multiple sub-centimeter (ranging 5 to 9 mm) hypodensities in the liver and kidney of uncertain significance. WAIHA often precedes the development of lymphoid malignancies, sometimes by several months; a repeat CT scan is planned in 2-3 months to reassess the abnormalities.

Graves’ disease is associated in some patients with autoimmune dysfunction of multiple organs, including pernicious anemia, type 1 diabetes mellitus, autoimmune adrenal insufficiency, vitiligo, systemic sclerosis, myasthenia gravis, Sjogren’s syndrome, systemic lupus erythematosis, and rheumatoid arthritis. Multiple cases of autoimmune hemolytic anemia associated with hyperthyroidism have been reported. In Graves’ disease, IgG type autoantibodies bind to the thyroid cell and activate the thyroid stimulating hormone receptor, which increases thyroid metabolic activity. In one case of hyperthyroidism with AIHA, treatment of hyperthyroidism with propylthiouracil not only restored euthyroidism but ameliorated the hemolytic anemia as well, without use of corticosteroids or blood transfusions [8]. The authors speculate that the hyperdynamic circulatory state in hyperthyroidism might accelerate hemolysis in antibody-coated red blood cells. Thomson et al. describe a patient who developed Graves’ disease during long-term immunosuppressive therapy for acquired autoimmune hemolytic anemia [10]. Thomson’s conclusion was that the IgG type immunoglobulins that cause Graves’ disease can develop even in the setting of long-term immunosuppressive treatment. At the time he developed WAIHA, our patient was clinically and chemically euthyroid after 18 months of propylthiouracil treatment. He had also been on immunosuppressive treatment with the tumor necrosis factor-inhibitor etanercept for several years, with good control of his reactive arthritis symptoms. The WAIHA seems not to have developed in response to a flare-up of either condition.

Reactive arthritis occurs after primary extraarticular infections, either enteric or sexually transmitted, and is characterized by the presence of bacterial antigen and/or viable but non-culturable bacteria that persist within the joint. HLA-B27 is present in 70-80% of patients with reactive arthritis, and HLA-B27 increases the risk of reactive arthritis by 25-fold. Although there are a number of known bacterial triggers for development of reactive arthritis, the precise mechanism of the pathologic host response is unknown. Possibilities include an HLA-B27 autoantigen, cellular uptake of causative organisms, toll-like receptors, and chemokine involvement [19]. Graves’ disease has been associated with a variety of infectious agents, including *Yersinia enterocolitica*, which is also one of the bacteria known to cause reactive arthritis [20].

*Yersinia enterocolitica* has not, however, been reported as a cause of WAIHA. We were unable to find any prior case reports of concurrent reactive arthritis and Graves disease. There is one report of a 30-year-old man in Papua New Guinea who developed severe hemolytic anemia (Hb 2.8) in the setting of reactive arthritis with urethritis, conjunctivitis, and keratoderma blenorrhagica [12]. Although a DAT was not reported, the author argued that the hemolysis was autoimmune because malaria and hypersplenism were excluded, and the anemia resolved with methotrexate and prednisolone treatment.

There are only a few case reports of hereditary autoimmune hemolytic anemia [13-16]. The paucity of reports and the sporadic pattern of involvement suggests that familial occurrence of AIHA is likely to be coincidental, although the sharing of HLA antigens 1 and 8 in a report of two affected sisters might argue for a genetic susceptibility in some cases [15]. It has been postulated that autoimmune diseases are a “mosaic” of genetic, immune, hormonal, and environmental factors. Genetic causes may include human leukocyte antigen (HLA) genes, non-HLA immune-regulatory or tissue-specific genes, and genetically-determined selective IgA deficiency [21]. Environmental factors may include infection, with Yersinia as a possibility, or the use of medications including long-term cytokine suppression with etanercept. A combination of genetic and environmental factors might have contributed to this patient’s development of multiple autoimmune diseases.

## Conclusion

We report the first case of concurrent reactive arthritis, Graves’ disease, and autoimmune hemolytic anemia. Yersinia enterocolitica infection could theoretically cause both reactive arthritis and Graves’ disease, although we cannot prove this connection in our patient’s case. It is not clear whether the autoimmune hemolysis was coincidental or related to an underlying condition that predisposed this patient to multiple autoimmune diseases. The family history of autoimmune hemolysis suggests the possibility of an inherited predisposition.

## Abbreviations

AIDS: autoimmune deficiency syndrome
CLL: chronic lymphocytic leukemia
DAT: Direct antiglobulin test
DIHA: drug-induced hemolytic anemia
Hb: hemoglobin
HLA: human leukocyte antigen
RA: rheumatoid arthritis
SLE: systemic lupus erythematosus
UC: ulcerative colitis
WAIHA: warm autoimmune hemolytic anemia
